# Primary versus secondary source of data in observational studies and heterogeneity in meta-analyses of drug effects: a survey of major medical journals

**DOI:** 10.1186/s12874-018-0561-3

**Published:** 2018-09-27

**Authors:** Guillermo Prada-Ramallal, Fatima Roque, Maria Teresa Herdeiro, Bahi Takkouche, Adolfo Figueiras

**Affiliations:** 1 Department of Preventive Medicine and Public Health, University of Santiago de Compostela, c/ San Francisco s/n, 15786 Santiago de Compostela, A Coruña, Spain; 2 Health Research Institute of Santiago de Compostela (Instituto de Investigación Sanitaria de Santiago de Compostela - IDIS), Clinical University Hospital of Santiago de Compostela, 15706 Santiago de Compostela, Spain; 3 Research Unit for Inland Development, Polytechnic of Guarda (Unidade de Investigação para o Desenvolvimento do Interior - UDI/IPG), 6300-559 Guarda, Portugal; 4 Health Sciences Research Centre, University of Beira Interior (Centro de Investigação em Ciências da Saúde - CICS/UBI), 6200-506 Covilhã, Portugal; 5 Department of Medical Sciences & Institute for Biomedicine – iBiMED, University of Aveiro, 3810-193 Aveiro, Portugal; 6 Higher Polytechnic & University Education Co-operative (Cooperativa de Ensino Superior Politécnico e Universitário - CESPU), Institute for Advanced Research & Training in Health Sciences & Technologies, 4585-116 Gandra, Portugal; 7 Consortium for Biomedical Research in Epidemiology & Public Health (CIBER en Epidemiología y Salud Pública – CIBERESP), Santiago de Compostela, Spain

**Keywords:** Observational studies, Meta-analysis, Source of data, Heterogeneity, Drug, Over-the-counter, Out-of-pocket

## Abstract

**Background:**

The data from individual observational studies included in meta-analyses of drug effects are collected either from ad hoc methods (i.e. “primary data”) or databases that were established for non-research purposes (i.e. “secondary data”). The use of secondary sources may be prone to measurement bias and confounding due to over-the-counter and out-of-pocket drug consumption, or non-adherence to treatment. In fact, it has been noted that failing to consider the origin of the data as a potential cause of heterogeneity may change the conclusions of a meta-analysis. We aimed to assess to what extent the origin of data is explored as a source of heterogeneity in meta-analyses of observational studies.

**Methods:**

We searched for meta-analyses of drugs effects published between 2012 and 2018 in general and internal medicine journals with an impact factor > 15. We evaluated, when reported, the type of data source (primary vs secondary) used in the individual observational studies included in each meta-analysis, and the exposure- and outcome-related variables included in sensitivity, subgroup or meta-regression analyses.

**Results:**

We found 217 articles, 23 of which fulfilled our eligibility criteria. Eight meta-analyses (8/23, 34.8%) reported the source of data. Three meta-analyses (3/23, 13.0%) included the method of outcome assessment as a variable in the analysis of heterogeneity, and only one compared and discussed the results considering the different sources of data (primary vs secondary).

**Conclusions:**

In meta-analyses of drug effects published in seven high impact general medicine journals, the origin of the data, either primary or secondary, is underexplored as a source of heterogeneity.

**Electronic supplementary material:**

The online version of this article (10.1186/s12874-018-0561-3) contains supplementary material, which is available to authorized users.

## Background

Specific research questions are ideally answered through tailor-made studies. Although these ad hoc studies provide more accurate and updated data, designing a completely new project may not represent a feasible strategy [1, 2]. On the other hand, clinical and administrative databases used for billing and other fiscal purposes (i.e. “secondary data”) are a valuable resource as an alternative to ad hoc methods (i.e. “primary data”) since it is easier and less costly to reuse the information than collecting it anew [3]. The potential of secondary automated databases for observational epidemiological studies is widely acknowledged; however, their use is not without challenges, and many quality requirements and methodological pitfalls must be considered [4].

Meta-analysis represents one of the most valuable tools for assessing drug effects as it may lead to the best evidence possible in epidemiology [5]. Consequently, its use for making relevant clinical and regulatory decisions on the safety and efficacy of drugs is dramatically increasing [6]. Existence of heterogeneity in a given meta-analysis is a feature that needs to be carefully described by analyzing the possible factors responsible for generating it [7]. In this regard, the results of a recent study [8] show that whether the origin of the data (primary vs secondary) is explored as a potential cause of heterogeneity may change the conclusions of a meta-analysis due to an effect modification [9]. Thus, considering the source of data as a variable in sensitivity and subgroup analyses, or meta-regression analyses, seems crucial to avoid misleading conclusions in meta-analyses of drug effects.

Given the evidence noted [8, 9], we surveyed published meta-analyses in a selection of high-impact journals over a 6-year period, to assess to what extent the origin of the data, either primary or secondary, is explored as a source of heterogeneity in meta-analyses of observational studies.

## Methods

### Meta-analysis selection and data collection process

General and internal medicine journals with an impact factor > 15 according to the Web of Science were included in the survey [10]. This method has been widely used to assess quality as well as publication trends in medical journals [11–13]. The rationale is that meta-analyses published in high impact journals: (1) are likely to be rigorously performed and reported due to the exhaustive editorial process [12, 14]; and, (2) in general, exert a higher influence on medical practice due to the major role played by these journals in the dissemination of the new medical evidence [14, 15]. We searched MEDLINE on May 2018 using the search terms “meta-analysis” as publication type and “drug” in any field between January 1, 2012 and May 7, 2018 in *the New England Journal of Medicine* (*NEJM*), *Lancet, Journal of the American Medical Association* (*JAMA)*, *British Medical Journal* (*BMJ*), *JAMA Internal Medicine (JAMA Intern Med)*, *Annals of Internal Medicine* (*Ann Intern Med*), and *Nature Reviews Disease Primers (Nat Rev Dis Primers)*.

Two investigators (GP-R, FR) independently assessed publications for eligibility. Abstracts were screened and if deemed potentially relevant, full text articles were retrieved. Articles were excluded if they met any of the following conditions: (1) were not a meta-analysis of published studies, (2) no drug effects were evaluated, (3) only randomized clinical trials were included in the meta-analysis (in order to consider observational studies), (4) less than two observational studies were included in the meta-analysis (since with a single study it would not have been possible to calculate a pooled measure). When a meta-analysis included both observational studies and clinical trials, only observational studies were considered.

A data extraction form was developed previously to extract information from articles. Two investigators (GP-R, FR) independently extracted and recorded the information and resolved discrepancies by referring to the original report. If necessary, a third author (AF) was asked to resolve disagreements between the investigators.

When available we extracted the following data from each eligible meta-analysis: first author, publication year, journal, drug(s) exposure and outcome(s); number of individual studies included in the meta-analysis based on each type of data source used (primary vs secondary), for both exposure and outcome assessment; and exposure- and outcome-related variables included in sensitivity, subgroup or meta-regression analyses. We extracted data directly from the tables, figures, text, and supplementary material of the meta-analyses, not from the individual studies.

### Assessment of exposure and outcome

We considered “primary data” the information on drug exposure collected directly by the researchers using interviews –personal or by telephone– or self-administered questionnaires. The origin of the data was also considered primary when objective diagnostic methods were used for the determination of drug exposure (e.g. blood test). “Secondary data” are data that were formerly collected for other purposes than that of the study at hand and that were included in databases on drug prescription (e.g. prescription registers, medical records/charts) and dispensing (e.g. computerized pharmacy records, insurance claims databases). Regarding the outcome assessment, we considered primary data when an objective confirmation is available that endorses them (e.g. confirmed by individual medical ad hoc diagnosis, lab test or imaging results). These criteria are based on those commonly used in the risk assessment of bias for observational studies [16–19].

## Results

**Fig. 1:**
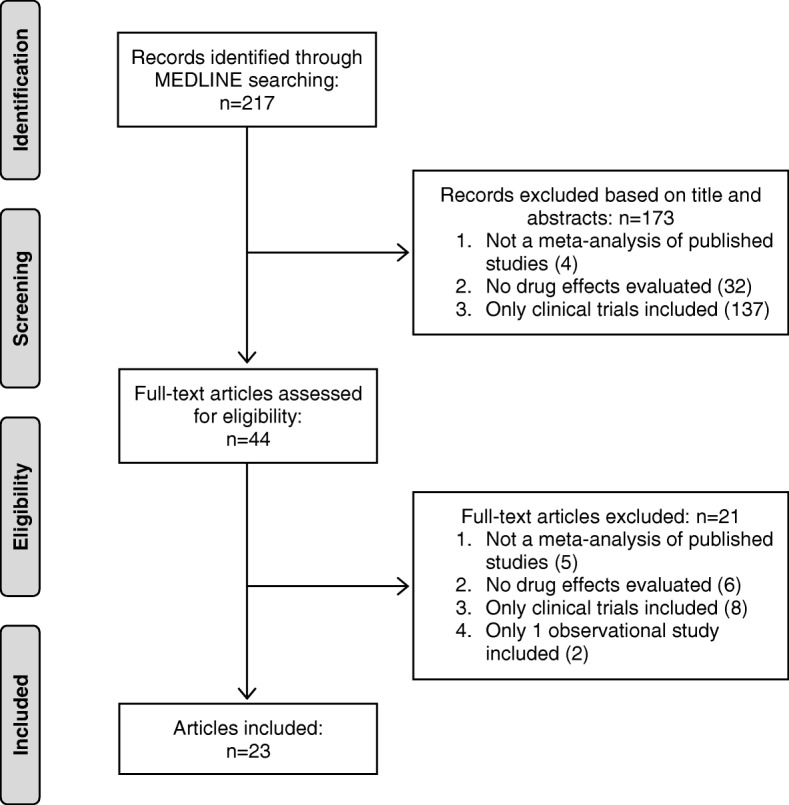
Flow diagram of literature search results

MEDLINE search results yielded 217 articles from the major general medical journals (3 from *NEJM*, 46 from *Lancet*, 26 from *JAMA*, 85 from *BMJ*, 19 from *JAMA Intern Med,* 38 from *Ann Intern Med,* and 0 from *Nat Rev Dis Primers*) (see Fig. 1). A total of 194 articles were excluded (see list of excluded articles with reasons for exclusion in Additional file 1) leaving 23 articles to be examined [20–42]. General characteristics of the 23 included meta-analyses are outlined in Table 1.

**Table 1:** Characteristics of the 23 included meta-analyses

Meta-analysis			Variables	
First author	Year	Journal	Drug exposure	Outcome
Weiss J [20]	2017	Ann Intern Med	Antihypertensive drugs	Harms outcomes: Cognitive impairment, quality of life, falls, fractures, syncope, functional status, hypotension, acute kidney injury, medication burden, withdrawal due to adverse events
Bally M [21]	2017	BMJ	NSAIDs	Myocardial infarction
Sordo L [22]	2017	BMJ	Opioid substitution treatment (methadone, buprenorphine)	All cause and overdose mortality
Tariq R [23]	2017	JAMA Intern Med	Gastric acid suppressants	Recurrent *Clostridium difficile* infection
Maruthur NM [24]	2016	Ann Intern Med	Diabetes monotherapy (thiazolidinediones, metformin, sulfonylureas, DPP-4 inhibitors, SGLT-2 inhibitors, GLP-1 receptor agonists) or metformin-based combinations	All-cause mortality, macrovascular and microvascular outcomes, intermediate outcomes (hemoglobin A1c, body weight, systolic blood pressure, heart rate), hypoglycemia, gastrointestinal side effects, genital mycotic infections, congestive heart failure
Paul S [25]	2016	Ann Intern Med	Antiviral prophylaxis	Primary outcome: Hepatitis B Virus (HBV) reactivation Secondary outcomes: HBV-related hepatitis, interrupted chemotherapy, acute liver failure, mortality
Li L [26]	2016	BMJ	Dipeptidyl peptidase-4 inhibitors	Heart failure
				Hospital admissions for heart failure
Molnar AO [27]	2015	BMJ	Generic immunosuppressive drugs	Patient survival, allograft survival, acute rejection, adverse events, bioequivalence
Ziff OJ [28]	2015	BMJ	Digoxin	Primary outcome: All-cause mortality
				Secondary outcomes: Cardiovascular mortality; admission to hospital for any cause, cardiovascular causes and heart failure; incident stroke, incident myocardial infarction
CGESOC [29]	2015	Lancet	Hormone therapy (oestrogen, progestagen)	Ovarian cancer
Bellemain- Appaix A [30]	2014	BMJ	Tienopyridines (clopidogrel)	Primary outcome: All-cause mortality, major bleeding Secondary outcomes: Major cardiovascular events and myocardial infarction, stroke, urgent revascularization, stent thrombosis
Grigoriadis S [31]	2014	BMJ	Antidepressants (SSRIs)	Persistent pulmonary hypertension of the newborn
Li L [32]	2014	BMJ	Incretin-based treatments	Pancreatitis
Kalil AC [33]	2014	JAMA	Vancomycin MIC	All-cause mortality
Stegeman BH [34]	2013	BMJ	Combined oral contraceptives	Venous thrombosis
Maneiro JR [35]	2013	JAMA Intern Med	Biologic agents (abatacept, adalimumab, etanercept, golimumab, infliximab, rituximab)	Influence of AABs: on efficacy in immune-mediated inflammatory diseases (rheumatoid arthritis, juvenile idiopathic arthritis, inflammatory bowel disease, ankylosing spondylitis, psoriasis, psoriatic arthritis, or other spondyloarthropathies), in hypersensitivity reactions, and on the concentration of biological drugs; effect of concomitant treatment in development of AAB
Hartling L [36]	2012	Ann Intern Med	Antipsychotics	Primary outcomes: Improved core symptoms of illness (positive and negative symptoms and general psychopathology), adverse events: diabetes mellitus, death, tardive dyskinesia, major metabolic syndrome
				Secondary outcomes: Functional outcomes, health care system use; response, remission and relapse rates; medication adherence, health-related quality of life, other patient-oriented outcomes (e.g. patient satisfaction), other adverse events: extrapyramidal symptoms, weight gain
Hsu J [37]	2012	Ann Intern Med	Antivirals (oseltamivir, zanamivir, amantadine, rimantadine)	Mortality, hospitalization, intensive care unit admission, mechanical ventilation and respiratory failure, duration of hospitalization, duration of signs and symptoms, time to return to normal activity, complications, critical adverse events: major psychotic disorders, encephalitis, stroke, seizure; important adverse events: pain in extremities, clonic twitching, body weakness, dermatologic changes (urticaria or rash); influenza viral shedding, emergence of antiviral resistance
Caldeira D [38]	2012	BMJ	ACEIs and ARBs	Incidence of pneumonia
				Pneumonia related mortality
MacArthur GJ [39]	2012	BMJ	Opiate substitution, methadone detoxification	HIV infection among people who inject drugs
Mantha S [40]	2012	BMJ	Progestin-only contaception	Venous thromboembolic events
Silvain J [41]	2012	BMJ	Enoxaparin, unfractioned heparin	Primary outcome: Mortality, major bleeding Secondary outcomes: Composite ischaemic end point (death or myocardial infarction), complications of myocardial infarction, minor bleeding
McKnight RF [42]	2012	Lancet	Lithium	Renal function, thyroid function, parathyroid function, hair disorders, skin disorders, bodyweight, teratogenicity

*Abbreviations*: *AABs* antibodies against biologic agents, *ACEIs*, angiotensin converting enzyme inhibitors, Ann Intern Med *Annals of Internal Medicine*, *ARBs* angiotensin receptor blockers, BMJ *British Medical Journal*, *DPP-4* Dipeptidyl Peptidase-4, *GLP-1* glucagon like peptide-1, JAMA *Journal of the American Medical Association*, *MIC* minimum inhibitory concentration, *NSAIDs* non-steroidal anti-inflammatory drugs, *SGLT-2* sodium–glucose cotransporter 2, *SSRIs* selective serotonin reuptake inhibitors

### Source of exposure and outcome data

Table 2 summarizes the evidence regarding the type of data source included in each meta-analysis, according to the information presented in the data extraction tables of the article. The information was evaluated taking the study design into account. Only eight meta-analyses [21, 24, 26, 31, 32, 34, 38, 41] reported the source of data, three of them [31, 34, 38] reporting mixed sources for both the exposure and outcome assessment. Five meta-analyses [21, 24, 26, 32, 41] reported only secondary sources for the exposure assessment, three of them [21, 24, 41] reporting as well only secondary sources for the outcome assessment, while in the other two [26, 32] only primary and mixed sources for the outcome assessment were reported respectively.

**Table 2:** Reporting of the data source in the data extraction tables of the included meta-analyses

Meta-analysis (MA)	Exposure assessment							Outcome assessment						
	Data source presented in MA	Cohort studies (n)			Case-control studies (n)			Data source presented in MA	Cohort studies (n)			Case-control studies (n)		
		1ry	2ry	NR	1ry	2ry	NR		1ry	2ry	NR	1ry	2ry	NR
Weiss J [20] Harms outcomes	No	.	.	.	.	.	.	No	.	.	.	.	.	.
Bally M [21]	Yes	0	3^b^	0	0	1	0	Yes	0	3^b^	0	0	1	0
Sordo L [22]	No^a^	.	.	.	.	.	.	No^a^	.	.	.	.	.	.
Tariq R [23]	No^ac^	.	.	.	.	.	.	No^a^	.	.	.	.	.	.
Maruthur NM [24]	Yes^d^	0	3	0	.	.	.	Yes^d^	0	3	0	.	.	.
Paul S [25]	No^a^	.	.	.	.	.	.	No^a^	.	.	.	.	.	.
Li L [26] Heart failure	Yes	0	1	2	0	0	1	Yes	1	0	2	0	0	1
Li L [26] Hospital admissions for heart failure	Yes	0	0	6	0	0	2	Yes	3	0	3	0	0	2
Molnar AO [27]	No^a^	.	.	.	.	.	.	No^a^	.	.	.	.	.	.
Ziff OJ [28]	No^a^	.	.	.	.	.	.	No^a^	.	.	.	.	.	.
CGESOC [29]	No	.	.	.	.	.	.	No	.	.	.	.	.	.
Bellemain-Appaix A [30]	No^a^	.	.	.	.	.	.	No^a^	.	.	.	.	.	.
Grigoriadis S [31]	Yes	2	3	0	1	1	0	Yes	4	1	0	2	0	0
Li L [32]	Yes	0	1	2	0	1	1	Yes	1	2	0	0	0	2
Kalil AC [33]	No	.	.	.	.	.	.	No	.	.	.	.	.	.
Stegeman BH [34]	Yes	0	9	0	8	8	1	Yes	4	5	0	5	12	0
Maneiro JR [35]	No^a^	.	.	.	.	.	.	No^a^	.	.	.	.	.	.
Hartling L [36]	No	.	.	.	.	.	.	No	.	.	.	.	.	.
Hsu J [37]	No^a^	.	.	.	.	.	.	No^a^	.	.	.	.	.	.
Caldeira D [38]	Yes	2	2	7	0	7	1	Yes	0	1	10	3	1	4
MacArthur GJ [39]	No^a^	.	.	.	.	.	.	No^a^	.	.	.	.	.	.
Mantha S [40]	No	.	.	.	.	.	.	No	.	.	.	.	.	.
Silvain J [41]	Yes	0	7	0	.	.	.	Yes	0	7	0	.	.	.
McKnight RF [42]	No	.	.	.	.	.	.	No	.	.	.	.	.	.

*Abbreviations*: *1ry* number of individual studies in each MA based on primary data sources, *2ry* number of individual studies in each MA based on secondary data sources, *NR* number of individual studies in each MA with not reported data source

^a^Although the meta-analysis shows the results of methodological quality assessment based on a standardized scale, it does not indicate the type of data source used for each individual observational study included in the meta-analysis

^b^Cohort with nested case-control analysis

^c^The meta-analysis reports that most of the included observational studies assessed medication exposure through a review of medical records

^d^The meta-analysis reports only data from high-quality observational studies

### Source of data in the analysis of heterogeneity

All but two [20, 42] of the meta-analyses performed subgroup and/or sensitivity analyses. Although three of them [23, 34, 36] considered the methods of outcome assessment – type of diagnostic assay used for *Clostridium difficile* infection, method of venous thrombosis diagnosis confirmation, and type of scale for psychosis symptoms assessment respectively– as stratification variables, only the second referred to the origin of the data. Only five meta-analyses [22, 28, 33, 35, 39] included meta-regression analyses to describe heterogeneity, none of which considered the source of data as an explanatory variable. Other findings for the inclusion of the data source as a variable in the analysis of heterogeneity are presented in Table 3.

**Table 3:** Inclusion of the data source as a variable in the analysis of heterogeneity of the included meta-analyses

Meta-analysis	Subgroup/ sensitivity analysis				Meta-regression analysis			
	Exposure-related variables	Outcome-related variables	Other variables	Type of data source included	Exposure-related variables	Outcome-related variables	Other variables	Type of data source included
Weiss J [20] Harms outcomes	.	.	.	No	.	.	.	No
Bally M [21]	Timing of exposure to NSAIDs, dosage and duration of treatment, concomitant drug treatment	Comorbidities	Alternative statistical model, reason for exclusion	No	.	.	.	No
Sordo L [22]	Time interval in and out of opioid substitution treatment	.	Alternative statistical model	No	Treatment provider, prevalence of opioid injection, average methadone dose	.	Mean age, percentage of men, location, percentage of inpatient induction, percentage loss to follow-up, midpoint follow-up period	No
Tariq R [23]	Type of gastric acid suppressant (PPI and H2B reported together, PPI alone, or H2B alone)	Case definition (time interval of recurrence: within 60 days vs within 90 days), type of diagnostic assay used for *Clostridium difficile* infection	Study design, study setting (inpatients vs outpatients), data adjustment	No	.	.	.	No
Maruthur NM [24]	Mode of therapy	.	.	No	.	.	.	No
Paul S [25] Primary outcome	.	Chronic or resolved hepatitis B virus infection	Tumor and chemotherapy subtype, alternative statistical model, quality of design	No	.	.	.	No
Paul S [25] Secondary outcomes	.	.	Alternative statistical model, quality of design	No	.	.	.	No
Li L [26]	Type of control, mode of therapy, individual drugs	.	Length of follow up, type of design	No	.	.	.	No
Molnar AO [27]	.	.	Type of design	No	.	.	.	No
Ziff OJ [28] Primary outcome	.	.	Data adjustment, population type	No	Difference between digoxin and control arms at baseline: Diabetes, hypertension, diuretics, anti-arrhythmic drugs	.	Summary bias score, baseline study level variable: Year of publication, age, sex, previous myocardial infarction	No
Ziff OJ [28] Secondary outcomes	.	.	.	No	.	.	.	No
CGESOC [29]	Duration of use in current and past users of hormone therapy, types of hormone therapy	Tumour histology and malignant potential of the tumour	Study design, geographical region, age at first use of hormone therapy, age at menarche, parity, oral contraceptive use, height, bosy mass index, alcohol use, tobacco use, mother or sister with ovarian/breast cancer, histerectomy	No	.	.	.	No
Bellemain-Appaix A [30]	Clopidogrel dose	Types of percutaneous coronary intervention	Type of design	No	.	.	.	No
Grigoriadis S [31]	Timing of exposure to SSRIs	.	Study design, congenital malformations, control, meconium aspiration	No	.	.	.	No
Li L [32]	Type of incretin agents, type of control, mode of therapy, individual incretin agents	.	Length of follow-up, alternative effect measure, alternative statistical model	No	.	.	.	No
Kalil AC [33]	Different MIC cutoffs, assay type	Hospital or 30-d mortality	Publication year, quality of design	No	Vancomycin MIC cut-off, vancomycin exposure in the previous 6 months, vancomycin trough levels, proportion of patients who received vancomycin treatment	Control mortality, APACHE II score, Charlson score, duration of bacteremia, proportion of patients with endocarditis, proportion of patients located in the intensive care unit	Age	No
Stegeman BH [34]	Generation of progestogen used in combined oral contraceptives, combined oral contraceptive pill	Method of diagnosis confirmation	Funding source, study design	Yes (outcome)	.	.	.	No
Maneiro JR [35]	Type of biologic agent, concomitant treatment (monotherapy vs combined therapy), prior use of TNF inhibitors	Type of disease	Length of follow-up, data quality, study design, level of evidence of studies	No	Type of biologic agent, prior use of TNF inhibitors, method of measurement of antibodies, type of the anti-TNF monoclonal antibody	Type of disease, time of disease duration, time to assess response	Age and sex of patients, number of participants, length of follow-up, data quality, study design, level of evidence of studies	No
Hartling L [36] Primary outcomes	Type of drug-comparison	Type of scale for the assessment of symptoms and quality of life	.	No	.	.	.	No
Hartling L [36] Secondary outcomes	.	.	.	No	.	.	.	No
Hsu J [37]	Individual drugs, dosage of antiviral, timing of treatment	.	Data adjustment, confirmed influenza, type of influenza A vs B, pandemic versus seasonal influenza, severity of influenza, age, pregnancy, baseline risk (e.g. immune-compromised), setting, funding conflict	No	.	.	.	No
Caldeira D [38] Incidence of pneumonia	.	.	Study design, previous stroke, heart failure, chronic kidney disease, non-Asian patients	No	.	.	.	No
Caldeira D [38] Pneumonia related mortality	.	.	Study design	No	.	.	.	No
MacArthur GJ [39]	Duration of exposure to opiate substitution treatment	.	Data adjustment, geographical region, site of recruitment, monetary incentives, percentage of female participants, percentage of individuals from ethnic minorities	No	Exposure to methadone maintenance treatment at baseline only	.	Inclusion only of studies at lower risk of bias, inclusion only of studies that measured an incidence rate ratio, exclusion of studies that did not adjust for confounders	No
Mantha S [40]	Route of administration	.	Data adjustment	No	.	.	.	No
Silvain J [41]	Route of administration	.	Types of percutaneous coronary intervention, study publication, study size, quality of design	No	.	.	.	No
McKnight RF [42]	.	.	.	No	.	.	.	No

*Abbreviations*: *APACHE* acute physiology and chronic health evaluation, *MIC* minimum inhibitory concentration, *SSRIs* selective serotonin reuptake inhibitors, *TNF* tumor necrosis factor

We finally assessed if the influence of the data origin on the conclusions of the meta-analyses was discussed by their respective authors. We found that only four meta-analyses [21, 31, 32, 34] noted limitations derived from the type of data source used.

## Discussion

The findings of this research suggest that the origin of the data, either primary or secondary, is *underexplored* as a source of heterogeneity and an effect modifier in meta-analyses of drug effects published in general medicine journals with high impact. Few meta-analyses reported the source of data and only one [34] of the articles included in our survey compared and discussed the meta-analysis results considering the different sources of data.

Although it is usual to consider the design of the individual studies (i.e. case-control, cohort or experimental studies) in the analysis of the heterogeneity of a meta-analysis [43, 44], the type of data source (primary vs secondary) is still rarely used for this purpose [9, 45]. In fact, the current reporting guidelines for meta-analyses, such as MOOSE (Meta-analysis Of Observational Studies in Epidemiology) [18] or PRISMA (Preferred Reporting Items for Systematic reviews and Meta-Analyses) [46, 47], do not recommend that authors specifically report the origin of the data. This is probably due to the close relationship that exists between the study design and the type of data source used, despite the fact that each criterion has its own basis. Performing this additional analysis is a simple task that involves no additional cost. Failure to do so may lead to *diverging conclusions* [8].

**Fig. 2:**
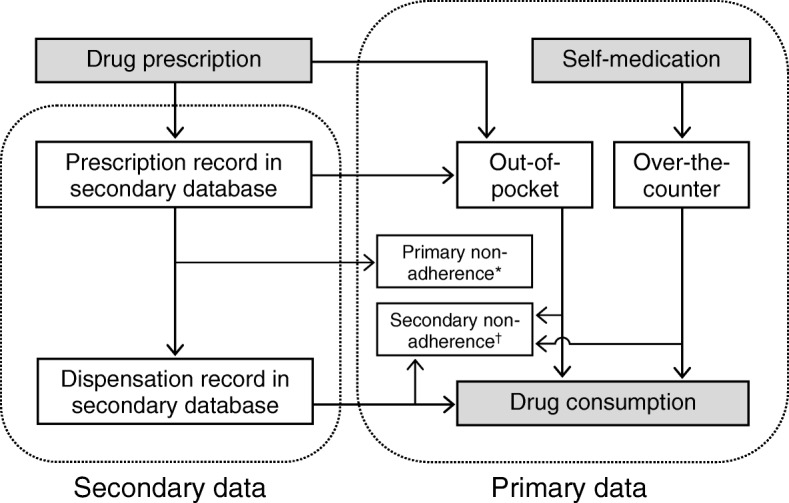
Conceptual model of individual data recording. ^*^ Never dispensed. ^†^ Absence of dispensing of successive prescriptions (or self-medication) among patients with primary adherence, or inadequate secondary adherence

Conclusions about the effects of a drug that are derived from studies based exclusively on data from secondary sources may be dicey, among other reasons, because no information is collected on consumption of *over-the-counter* drugs (i.e. drugs that individuals can buy without a prescription) [48] and/or *out-of-pocket* expenses for prescription drugs (i.e. costs that individuals pay out of their own cash reserves) [49]. In the health care and insurance context, out-of-pocket expenses usually refer to deductibles, co-payments or co-insurance. Figure 2 shows the model that we propose to describe the relationship between the different data records according to their origin, including the possible loss of information (susceptible to be registered only through primary research).

Failure to take these situations into account may lead to *exposure measurement bias* [48, 49]. Consumption of a drug may be underestimated when only prescription data is used as secondary source without additionally considering unregistered consumption, such as over-the-counter consumption (e.g. oral contraceptives [34, 50]), that may only be available from a primary database. Alternatively, this may occur when dispensing data for billing purposes (reimbursement) are used for clinical research, if out-of-pocket expenses are not considered (see Fig. 2). The portion of the medical bill that the insurance company does not cover, and that the individual must pay on his own, is unlikely to be recorded. Data on the sale of over-the-counter drugs will also not be available in this scenario.

The reverse situation may also occur and consumption may be overestimated when only prescription data is used, if the prescribed drug is not dispensed by the pharmacist; or when dispensing data is used, if the drug is not really consumed by the patient. While primary non-adherence occurs when the patient does not pick up the medication after the first prescription, secondary non-adherence refers to the absence of dispensing of successive prescriptions among patients with primary adherence, or to inadequate secondary adherence (i.e. ≥20% of time without adequate medication) [51] (see Fig. 2). In some diseases the medication adherence is very low [52–55], with percentages of primary non-adherence (never dispensed) that exceed 30% [56]. It should be noted that the impact of non-adherence varies from medication to medication. Therefore, it must be defined and measured in the context of a particular therapy [57].

Moreover, failing to take into consideration the portion of consumption due to over-the-counter and/or out-of-pocket expenses may lead to *confounding*, as that variable may be related to the socio-economic level and/or to the potential of access to the health system [58], which are independent risk factors of adverse outcomes of some medications (e.g. myocardial infarction [21, 28, 30, 41]). Given the presence of high-deductible health plans and the high co-insurance rate for some drugs, cost-sharing may deter clinically vulnerable patients from initiating essential medications, thus negatively affecting patient adherence [59, 60].

*Outcome misclassification* may also give rise to measurement bias and heterogeneity [61]. This occurs, for example, in the meta-analysis that evaluates the relationship between combined oral contraceptives and the risk of venous thrombosis [34]. In the studies without objective confirmation of the outcome, the women were classified erroneously regardless of the use of contraceptives. This led to a non-differential misclassification that may have underestimated the drug–outcome relationship, especially when the third generation of progestogen is analysed: Risk ratio (RR) primary data = 6.2 (95% confidence interval (CI) 5.2–7.4), RR secondary data = 3.0 (95% CI 1.7–5.4) [34].

On the one hand, medical records are often considered as being the best information source for outcome variables. However, they present important limitations in the recording of medications taken by patients [62]. On the other hand, dispensing records show more detailed data on the measurement of drug exposure. However, they do not record the over-the-counter or out-of-pocket drug consumption at an individual level [48, 49], apart from offering unreliable data on outcome variables [62, 63].

### Limitations

The first limitation of this research is that its findings may not be applicable to journals not included in our survey such as journals with low impact factor. Despite the widespread use of the *impact factor metric* [64], this method has inherent weaknesses [65, 66]. However, meta-analyses published in high impact general medicine journals are likely to be most rigorously performed and reported due to their greater availability of resources and procedures [12, 14]. It is then expected that the overall reporting quality of articles published in other lesser-known journals will be similar. Another limitation would be related to the limited *search period*. In this sense, and given that the general tendency is the improvement of the methodology of published meta-analyses [67, 68], we find no reason to suspect that the adverse conclusions could be different before the period from 2012 to 2018. Although it exceeds the objective of this research, one last limitation may be the inability to reanalyse the included meta-analyses stratifying by the type of data source since our study design restricts the conclusions to the published data of the meta-analyses, which were *insufficiently reported*, or the number of individual studies in each stratum was insufficient to calculate a pooled measure (see Table 2).

## Conclusions

Owing to automated capture of data on drug prescription and dispensing that are used for billing and other administration purposes, as well as to the implementation of electronic medical records, secondary databases have generated enormous possibilities. However, neither their limitations, nor the risk of bias that they pose should be overlooked [69]. Thus, researchers should consider the link between administrative databases and medical records, as well as the advisability of *combining* secondary and primary data in order to minimize the occurrence of biases due to the use of any of these databases.

No source of heterogeneity in a meta-analysis should ever be considered alone but always as part of an interconnected set of potential questions to be addressed. In particular, the origin of the data, either primary or secondary, is insufficiently explored as a source of heterogeneity in meta-analyses of drug effects, even in those published in high impact general medicine journals. Thus, we believe that authors should *systematically* include the source of data as an additional variable in subgroup and sensitivity analyses, or meta-regression analyses, and discuss its influence on the meta-analysis results. Likewise, reviewers, editors and future guidelines should also consider the origin of the data as a potential cause of heterogeneity in meta-analyses of observational studies that include both primary and secondary data. Failure to do this may lead to misleading conclusions, with negative effects on clinical and regulatory decisions.

## Additional file

Additional file 1: Excluded articles. List of articles excluded with reasons for exclusion. (PDF 247 kb)

## Abbreviations

Ann Intern Med: Annals of Internal Medicine
BMJ: British Medical Journal
CI: Confidence Interval
JAMA Intern Med: JAMA Internal Medicine
JAMA: Journal of the American Medical Association
MOOSE: Meta-analysis Of Observational Studies in Epidemiology
Nat Rev Dis Primers: Nature Reviews Disease Primers
NEJM: New England Journal of Medicine
PRISMA: Preferred Reporting Items for Systematic reviews and Meta-Analyses
RR: Risk ratio
VS: Versus
