# Supplementary material for: Primary versus secondary source of data in observational studies and heterogeneity in meta-analyses of drug effects: a survey of major medical journals
Source: BMC Med Res Methodol. 2018 Sep 27;18:97. doi: 10.1186/s12874-018-0561-3 (PMC6161342; doi:10.1186/s12874-018-0561-3)
Supplement: Supplementary file 1 — Excluded articles. List of articles excluded with reasons for exclusion. (PDF 247 kb) [file 12874_2018_561_MOESM1_ESM.pdf]

Additional file 1

Excluded articles with reasons for exclusion

First author	Year	Journal	Reason for exclusion
Baxi S [1]	2018	BMJ	3. Only clinical trials included
Navarese EP [2]	2018	JAMA	3. Only clinical trials included
Zheng SL [3]	2018	JAMA	3. Only clinical trials included
Mitra S [4]	2018	JAMA	3. Only clinical trials included
Sobieraj DM [5]	2018	JAMA	3. Only clinical trials included
Sobieraj DM [6]	2018	JAMA	3. Only clinical trials included
Gayet-Ageron A [7]	2018	Lancet	3. Only clinical trials included
Zhang XL [8]	2017	Ann Intern Med	2. No drug effects evaluated
Alibhai SMH [9]	2017	Ann Intern Med	3. Only clinical trials included
Zenner D [10]	2017	Ann Intern Med	3. Only clinical trials included
Wilson LM [11]	2017	Ann Intern Med	3. Only clinical trials included
Desai M [12]	2017	BMJ	1. Not a meta-analysis of published studies
López-López JA [13]	2017	BMJ	3. Only clinical trials included
Croles FN [14]	2017	BMJ	2. No drug effects evaluated
Moser W [15]	2017	BMJ	3. Only clinical trials included
Sadeghirad B [16]	2017	BMJ	3. Only clinical trials included
Batelaan NM [17]	2017	BMJ	3. Only clinical trials included
Liu J [18]	2017	BMJ	3. Only clinical trials included
Bangalore S [19]	2017	BMJ	3. Only clinical trials included
Zhao JG [20]	2017	JAMA	3. Only clinical trials included
Tasillo A [21]	2017	JAMA Intern Med	1. Not a meta-analysis of published studies
Malhotra R [22]	2017	JAMA Intern Med	3. Only clinical trials included
Tsai WC [23]	2017	JAMA Intern Med	3. Only clinical trials included
Musso G [24]	2017	JAMA Intern Med	3. Only clinical trials included
Pan H [25]	2017	N Engl J Med	3. Only clinical trials included
Brownley KA [26]	2016	Ann Intern Med	3. Only clinical trials included
Collister D [27]	2016	Ann Intern Med	3. Only clinical trials included
Zaccardi F [28]	2016	Ann Intern Med	3. Only clinical trials included
Zhang XL [29]	2016	Ann Intern Med	2. No drug effects evaluated
[No author] [30]	2016	BMJ	1. Not a meta-analysis of published studies
Bangalore S [31]	2016	BMJ	3. Only clinical trials included
Boyle RJ [32]	2016	BMJ	2. No drug effects evaluated
Brunström M [33]	2016	BMJ	3. Only clinical trials included
Bryce A [34]	2016	BMJ	2. No drug effects evaluated
Gargiulo G [35]	2016	BMJ	3. Only clinical trials included
Hazlewood GS [36]	2016	BMJ	3. Only clinical trials included
Hollingsworth JM [37]	2016	BMJ	3. Only clinical trials included
Kotecha D [38]	2016	BMJ	3. Only clinical trials included
Livingstone KM [39]	2016	BMJ	3. Only clinical trials included
Salvo F [40]	2016	BMJ	3. Only clinical trials included
Sharma T [41]	2016	BMJ	3. Only clinical trials included

Chou R [42]	2016	JAMA	3. Only clinical trials included
Franco OH [43]	2016	JAMA	2. No drug effects evaluated
Khera R [44]	2016	JAMA	3. Only clinical trials included
Lotta LA [45]	2016	JAMA	2. No drug effects evaluated
Palmer SC [46]	2016	JAMA	3. Only clinical trials included
Silverman MG [47]	2016	JAMA	3. Only clinical trials included
Abdel Shaheed C [48]	2016	JAMA Intern Med	3. Only clinical trials included
Jaspers L [49]	2016	JAMA Intern Med	3. Only clinical trials included
Thakkar J [50]	2016	JAMA Intern Med	2. No drug effects evaluated
Cassese S [51]	2016	Lancet	2. No drug effects evaluated
Cipriani A [52]	2016	Lancet	3. Only clinical trials included
da Costa BR [53]	2016	Lancet	3. Only clinical trials included
Ettehad D [54]	2016	Lancet	3. Only clinical trials included
NCD-RisF [55]	2016	Lancet	2. No drug effects evaluated
Rothwell PM [56]	2016	Lancet	3. Only clinical trials included
Stone GW [57]	2016	Lancet	2. No drug effects evaluated
Xie X [58]	2016	Lancet	3. Only clinical trials included
Bannuru RR [59]	2015	Ann Intern Med	3. Only clinical trials included
Bannuru RR [60]	2015	Ann Intern Med	3. Only clinical trials included
Chou R [61]	2015	Ann Intern Med	3. Only clinical trials included
Miligkos M [62]	2015	Ann Intern Med	3. Only clinical trials included
Navarese EP [63]	2015	Ann Intern Med	3. Only clinical trials included
Siemieniuk RA [64]	2015	Ann Intern Med	3. Only clinical trials included
Smith ME [65]	2015	Ann Intern Med	3. Only clinical trials included
Spencer FA [66]	2015	Ann Intern Med	3. Only clinical trials included
Sundström J [67]	2015	Ann Intern Med	3. Only clinical trials included
Uthman OA [68]	2015	Ann Intern Med	3. Only clinical trials included
Alfirevic Z [69]	2015	BMJ	3. Only clinical trials included
Balsells M [70]	2015	BMJ	3. Only clinical trials included
Bolland MJ [71]	2015	BMJ	2. No drug effects evaluated
Conway R [72]	2015	BMJ	3. Only clinical trials included
Emanuel EJ [73]	2015	BMJ	1. Not a meta-analysis of published studies
Giacoppo D [74]	2015	BMJ	2. No drug effects evaluated
Lenzer J [75]	2015	BMJ	1. Not a meta-analysis of published studies
Li BZ [76]	2015	BMJ	3. Only clinical trials included
Machado GC [77]	2015	BMJ	3. Only clinical trials included
Navarese EP [78]	2015	BMJ	3. Only clinical trials included
Thomas KH [79]	2015	BMJ	3. Only clinical trials included
Emdin CA [80]	2015	JAMA	3. Only clinical trials included
Whiting PF [81]	2015	JAMA	3. Only clinical trials included
Di Bona D [82]	2015	JAMA Intern Med	3. Only clinical trials included
Prasad V [83]	2015	JAMA Intern Med	1. Not a meta-analysis of published studies
Viswanathan M [84]	2015	JAMA Intern Med	2. No drug effects evaluated
BPLTTC [85]	2015	Lancet	3. Only clinical trials included
Dobson J [86]	2015	Lancet	3. Only clinical trials included
EBCTCG [87]	2015	Lancet	3. Only clinical trials included

EBCTCG [88]	2015	Lancet	3. Only clinical trials included
Elmariah S [89]	2015	Lancet	3. Only clinical trials included
Hole J [90]	2015	Lancet	2. No drug effects evaluated
Mega JL [91]	2015	Lancet	4. Only 1 observational study included
Palmer SC [92]	2015	Lancet	3. Only clinical trials included
Palmerini T [93]	2015	Lancet	3. Only clinical trials included
Singh JA [94]	2015	Lancet	3. Only clinical trials included
Siontis GC [95]	2015	Lancet	2. No drug effects evaluated
Swerdlow DI [96]	2015	Lancet	3. Only clinical trials included
Viale L [97]	2015	Lancet	2. No drug effects evaluated
Al-Khatib SM [98]	2014	Ann Intern Med	3. Only clinical trials included
Danese S [99]	2014	Ann Intern Med	3. Only clinical trials included
Griebeler ML [100]	2014	Ann Intern Med	3. Only clinical trials included
Shaw RJ [101]	2014	Ann Intern Med	2. No drug effects evaluated
Stagg HR [102]	2014	Ann Intern Med	3. Only clinical trials included
Tu B [103]	2014	Ann Intern Med	2. No drug effects evaluated
Bramham K [104]	2014	BMJ	2. No drug effects evaluated
Ford AC [105]	2014	BMJ	3. Only clinical trials included
Gough EK [106]	2014	BMJ	3. Only clinical trials included
Jefferson [107]	2014	BMJ	3. Only clinical trials included
Keene D [108]	2014	BMJ	3. Only clinical trials included
Knoll GA [109]	2014	BMJ	3. Only clinical trials included
Loymans RJ [110]	2014	BMJ	3. Only clinical trials included
Naci H [111]	2014	BMJ	3. Only clinical trials included
Taylor D [112]	2014	BMJ	3. Only clinical trials included
Tricco AC [113]	2014	BMJ	4. Only 1 observational study included
Valgimigli M [114]	2014	BMJ	2. No drug effects evaluated
Windecker S [115]	2014	BMJ	3. Only clinical trials included
Coresh J [116]	2014	JAMA	2. No drug effects evaluated
Castellucci LA [117]	2014	JAMA	3. Only clinical trials included
Chatterjee S [118]	2014	JAMA	3. Only clinical trials included
Jonas DE [119]	2014	JAMA	3. Only clinical trials included
Lee JK [120]	2014	JAMA	3. Only clinical trials included
Sachar H [121]	2014	JAMA Intern Med	3. Only clinical trials included
Zusterzeel R [122]	2014	JAMA Intern Med	3. Only clinical trials included
Cheng J [123]	2014	JAMA Intern Med	3. Only clinical trials included
Sipahi I [124]	2014	JAMA Intern Med	3. Only clinical trials included
Sipahi I [125]	2014	JAMA Intern Med	3. Only clinical trials included
BPLTTC [126]	2014	Lancet	3. Only clinical trials included
Cortazar P [127]	2014	Lancet	3. Only clinical trials included
Emberson J [128]	2014	Lancet	3. Only clinical trials included
Eng C [129]	2014	Lancet	3. Only clinical trials included
Kotecha D [130]	2014	Lancet	3. Only clinical trials included
NSCLC [131]	2014	Lancet	3. Only clinical trials included
Reid IR [132]	2014	Lancet	2. No drug effects evaluated
Ruff CT [133]	2014	Lancet	3. Only clinical trials included

D'Ambrosio L [134]	2014	NEJM	1. Not a meta-analysis of published studies
Fu R [135]	2013	Ann Intern Med	3. Only clinical trials included
Lee M [136]	2013	Ann Intern Med	3. Only clinical trials included
Morgan RL [137]	2013	Ann Intern Med	2. No drug effects evaluated
Raman G [138]	2013	Ann Intern Med	2. No drug effects evaluated
Vasilakou D [139]	2013	Ann Intern Med	3. Only clinical trials included
Azad MB [140]	2013	BMJ	2. No drug effects evaluated
Bangalore S [141]	2013	BMJ	2. No drug effects evaluated
Bermingham SL [142]	2013	BMJ	2. No drug effects evaluated
BPLTTC [143]	2013	BMJ	3. Only clinical trials included
Chatterjee S [144]	2013	BMJ	3. Only clinical trials included
Cipriani A [145]	2013	BMJ	3. Only clinical trials included
Dormuth CR [146]	2013	BMJ	1. Not a meta-analysis of published studies
Gagne JJ [147]	2013	BMJ	3. Only clinical trials included
Gatta L [148]	2013	BMJ	3. Only clinical trials included
Makani H [149]	2013	BMJ	3. Only clinical trials included
Marschall J [150]	2013	BMJ	3. Only clinical trials included
Naci H [151]	2013	BMJ	3. Only clinical trials included
Navarese EP [152]	2013	BMJ	2. No drug effects evaluated
Wu HY [153]	2013	BMJ	3. Only clinical trials included
Kayentao K [154]	2013	JAMA	3. Only clinical trials included
Mead GE [155]	2013	JAMA	3. Only clinical trials included
Meissner K [156]	2013	JAMA Intern Med	3. Only clinical trials included
Wilt TJ [157]	2013	JAMA Intern Med	3. Only clinical trials included
CNT [158]	2013	Lancet	3. Only clinical trials included
Cuzick J [159]	2013	Lancet	3. Only clinical trials included
Leucht S [160]	2013	Lancet	3. Only clinical trials included
Stefanini GG [161]	2013	Lancet	2. No drug effects evaluated
Tusting LS [162]	2013	Lancet	2. No drug effects evaluated
Johnston BC [163]	2012	Ann Intern Med	2. No drug effects evaluated
Pinto RZ [164]	2012	Ann Intern Med	3. Only clinical trials included
Rutjes AW [165]	2012	Ann Intern Med	3. Only clinical trials included
Yeh HC [166]	2012	Ann Intern Med	3. Only clinical trials included
Asbridge M [167]	2012	BMJ	2. No drug effects evaluated
Bangalore S [168]	2012	BMJ	2. No drug effects evaluated
Farmer AJ [169]	2012	BMJ	2. No drug effects evaluated
Fox BD [170]	2012	BMJ	3. Only clinical trials included
Fürst T [171]	2012	BMJ	1. Not a meta-analysis of published studies
Haas DM [172]	2012	BMJ	3. Only clinical trials included
Harel Z [173]	2012	BMJ	3. Only clinical trials included
Hemmingsen B [174]	2012	BMJ	3. Only clinical trials included
Huedo-Medina TB [175]	2012	BMJ	3. Only clinical trials included
Karagiannis T [176]	2012	BMJ	3. Only clinical trials included
Low EV [177]	2012	BMJ	3. Only clinical trials included
Pinto RZ [178]	2012	BMJ	3. Only clinical trials included
Prochaska JJ [179]	2012	BMJ	3. Only clinical trials included

Vilsbøll T [180]	2012	BMJ	3. Only clinical trials included
Boekholdt SM [181]	2012	JAMA	3. Only clinical trials included
Jackson JL [182]	2012	JAMA	3. Only clinical trials included
Lopez-Olivo MA [183]	2012	JAMA	3. Only clinical trials included
Preiss D [184]	2012	JAMA	3. Only clinical trials included
Crawley J [185]	2012	Lancet	3. Only clinical trials included
EBCTCG [186]	2012	Lancet	3. Only clinical trials included
Heneghan C [187]	2012	Lancet	2. No drug effects evaluated
IL6R MR Consortium [188]	2012	Lancet	3. Only clinical trials included
Leucht S [189]	2012	Lancet	3. Only clinical trials included
Palmerini T [190]	2012	Lancet	2. No drug effects evaluated
Rothwell PM [191]	2012	Lancet	3. Only clinical trials included
Thiele H [192]	2012	Lancet	3. Only clinical trials included
Tricco AC [193]	2012	Lancet	2. No drug effects evaluated
Bischoff-Ferrari HÁ [194]	2012	NEJM	3. Only clinical trials included

Abbreviations: Ann Intern Med, *Annals of Internal Medicine*; BMJ, *British Medical Journal*; JAMA, *Journal of the American Medical Association*; JAMA Intern Med, *JAMA Internal Medicine*; NEJM, *New England Journal of Medicine*.

References

1. Baxi S, Yang A, Gennarelli RL, et al. Immune-related adverse events for anti-PD-1 and anti-PD-L1 drugs: systematic review and meta-analysis. BMJ. 2018;360:k793.

2. Navarese EP, Robinson JG, Kowalewski M, et al. Association between baseline LDL-C level and total and cardiovascular mortality after LDL-C lowering: a systematic review and meta-analysis. JAMA. 2018;319:1566-79.

3. Zheng SL, Roddick AJ, Aghar-Jaffar R, et al. Association between use of sodium-glucose cotransporter 2 inhibitors, glucagon-like peptide 1 agonists, and dipeptidyl peptidase 4 inhibitors with all-cause mortality in patients with type 2 diabetes: a systematic review and meta-analysis. JAMA. 2018;319:1580-91.

4. Mitra S, Florez ID, Tamayo ME, et al. Association of placebo, indomethacin, ibuprofen, and acetaminophen with closure of hemodynamically significant patent ductus arteriosus in preterm infants: a systematic review and meta-analysis. JAMA. 2018;319:1221-38.

5. Sobieraj DM, Weeda ER, Nguyen E, et al. Association of inhaled corticosteroids and long-acting β-agonists as controller and quick relief therapy with exacerbations and symptom control in persistent asthma: a systematic review and meta-analysis. JAMA. 2018;319:1485-96.

6. Sobieraj DM, Baker WL, Nguyen E, et al. Association of inhaled corticosteroids and long-acting muscarinic antagonists with asthma control in patients with uncontrolled, persistent asthma: a systematic review and meta-analysis. JAMA. 2018;319:1473-84.

7. Gayet-Ageron A, Prieto-Merino D, Ker K, et al. Effect of treatment delay on the effectiveness and safety of antifibrinolytics in acute severe haemorrhage: a meta-analysis of individual patient-level data from 40 138 bleeding patients. Lancet. 2018;391:125-32.

8. Zhang XL, Zhu QQ, Kang LN, et al. Mid- and long-term outcome comparisons of everolimus-eluting bioresorbable scaffolds versus everolimus-eluting metallic stents: a systematic review and meta-analysis. Ann Intern Med. 2017;167:642-54.

9. Alibhai SMH, Zukotynski K, Walker-Dilks C, et al. Bone health and bone-targeted therapies for nonmetastatic prostate cancer: a systematic review and meta-analysis. Ann Intern Med. 2017;167:341-50.

10. Zenner D, Beer N, Harris RJ, et al. Treatment of latent tuberculosis infection: an updated network meta-analysis. Ann Intern Med. 2017;167:248-55.

11. Wilson LM, Rebholz CM, Jirru E, et al. Benefits and harms of osteoporosis medications in patients with chronic kidney disease: a systematic review and meta-analysis. Ann Intern Med. 2017;166:649-58.

12. Desai M, Field N, Grant R, McCormack S. Recent advances in pre-exposure prophylaxis for HIV. BMJ. 2017;359:j5011.

13. López-López JA, Sterne JAC, Thom HHZ, et al. Oral anticoagulants for prevention of stroke in atrial fibrillation: systematic review, network meta-analysis, and cost effectiveness analysis. BMJ. 2017;359:j5058. Erratum in: BMJ. 2017;359:j5631.

14. Croles FN, Nasserinejad K, Duvekot JJ, et al. Pregnancy, thrombophilia, and the risk of a first venous thrombosis: systematic review and bayesian meta-analysis. BMJ. 2017;359:j4452.

15. Moser W, Schindler C, Keiser J. Efficacy of recommended drugs against soil transmitted helminths: systematic review and network meta-analysis. BMJ. 2017;358:j4307.

16. Sadeghirad B, Siemieniuk RAC, Brignardello-Petersen R, et al. Corticosteroids for treatment of sore throat: systematic review and meta-analysis of randomised trials. BMJ. 2017;358:j3887.

17. Batelaan NM, Bosman RC, Muntingh A, et al. Risk of relapse after antidepressant discontinuation in anxiety disorders, obsessive-compulsive disorder, and post-traumatic stress disorder: systematic review and meta-analysis of relapse prevention trials. BMJ. 2017;358:j3927. Erratum in: BMJ. 2017;358:j4461.

18. Liu J, Li L, Deng K, et al. Incretin based treatments and mortality in patients with type 2 diabetes: systematic review and meta-analysis. BMJ. 2017;357:j2499.

19. Bangalore S, Fakheri R, Wandel S, et al. Renin angiotensin system inhibitors for patients with stable coronary artery disease without heart failure: systematic review and meta-analysis of randomized trials. BMJ. 2017;356:j4.

20. Zhao JG, Zeng XT, Wang J, et al. Association between calcium or vitamin d supplementation and fracture incidence in community-dwelling older adults: a systematic review and meta-analysis. JAMA. 2017;318:2466-82.

21. Tasillo A, Salomon JA, Trikalinos TA, et al. Cost-effectiveness of Testing and Treatment for Latent Tuberculosis Infection in Residents Born Outside the United States With and Without Medical Comorbidities in a Simulation Model. JAMA Intern Med. 2017;177:1755-64.

22. Malhotra R, Nguyen HA, Benavente O, et al. Association Between More Intensive vs Less Intensive Blood Pressure Lowering and Risk of Mortality in Chronic Kidney Disease Stages 3 to 5: A Systematic Review and Meta-analysis. JAMA Intern Med. 2017;177:1498-505.

23. Tsai WC, Wu HY, Peng YS, et al. Association of Intensive Blood Pressure Control and Kidney Disease Progression in Nondiabetic Patients With Chronic Kidney Disease: A Systematic Review and Meta-analysis. JAMA Intern Med. 2017;177:792-9.

24. Musso G, Cassader M, Paschetta E, et al. Thiazolidinediones and Advanced Liver Fibrosis in Nonalcoholic Steatohepatitis: A Meta-analysis. JAMA Intern Med. 2017;177:633-40.

25. Pan H, Gray R, Braybrooke J, et al. 20-year risks of breast-cancer recurrence after stopping endocrine therapy at 5 years. N Engl J Med. 2017;377:1836-46.

26. Brownley KA, Berkman ND, Peat CM, et al. Binge-eating disorder in adults: a systematic review and meta-analysis. Ann Intern Med. 2016;165:409-20.

27. Collister D, Komenda P, Hiebert B, et al. The effect of erythropoietin-stimulating agents on health-related quality of life in anemia of chronic kidney disease: a systematic review and meta-analysis. Ann Intern Med. 2016;164:472-8.

28. Zaccardi F, Htike ZZ, Webb DR, et al. Benefits and harms of once-weekly glucagon-like peptide-1 receptor agonist treatments: a systematic review and network meta-analysis. Ann Intern Med. 2016;164:102-13.

29. Zhang XL, Zhu L, Wei ZH, et al. Comparative efficacy and safety of everolimus-eluting bioresorbable scaffold versus everolimus-eluting metallic stents: a aystematic review and meta-analysis. Ann Intern Med. 2016;164:752-63.

30. Optimal duration of dual antiplatelet therapy after percutaneous coronary intervention with drug eluting stents: meta-analysis of randomised controlled trials. BMJ. 2016;355:i5600.

31. Bangalore S, Fakheri R, Toklu B, et al. Diabetes mellitus as a compelling indication for use of renin angiotensin system blockers: systematic review and meta-analysis of randomized trials. BMJ. 2016;352:i438. Erratum in: BMJ. 2016;352:i1525.

32. Boyle RJ, Ierodiakonou D, Khan T, et al. Hydrolysed formula and risk of allergic or autoimmune disease: systematic review and meta-analysis. BMJ. 2016;352:i974.

33. Brunström M, Carlberg B. Effect of antihypertensive treatment at different blood pressure levels in patients with diabetes mellitus: systematic review and meta-analyses. BMJ. 2016;352:i717.

34. Bryce A, Hay AD, Lane IF, et al. Global prevalence of antibiotic resistance in paediatric urinary tract infections caused by Escherichia coli and association with routine use of antibiotics in primary care: systematic review and meta-analysis. BMJ. 2016;352:i939.

35. Gargiulo G, Windecker S, da Costa BR, et al. Short term versus long term dual antiplatelet therapy after implantation of drug eluting stent in patients with or without diabetes: systematic review and meta-analysis of individual participant data from randomised trials. BMJ. 2016;355:i5483.

36. Hazlewood GS, Barnabe C, Tomlinson G, et al. Methotrexate monotherapy and methotrexate combination therapy with traditional and biologic disease modifying antirheumatic drugs for rheumatoid arthritis: abridged Cochrane systematic review and network meta-analysis. BMJ. 2016;353:i1777.

37. Hollingsworth JM, Canales BK, Rogers MA, et al. Alpha blockers for treatment of ureteric stones: systematic review and meta-analysis. BMJ. 2016;355:i6112.

38. Kotecha D, Manzano L, Krum H, et al. Effect of age and sex on efficacy and tolerability of β blockers in patients with heart failure with reduced ejection fraction: individual patient data meta-analysis. BMJ. 2016;353:i1855.

39. Livingstone KM, Celis-Morales C, Papandonatos GD, et al. FTO genotype and weight loss: systematic review and meta-analysis of 9563 individual participant data from eight randomised controlled trials. BMJ. 2016;354:i4707.

40. Salvo F, Moore N, Arnaud M, et al. Addition of dipeptidyl peptidase-4 inhibitors to sulphonylureas and risk of hypoglycaemia: systematic review and meta-analysis. BMJ. 2016;353:i2231.

41. Sharma T, Guski LS, Freund N, et al. Suicidality and aggression during antidepressant treatment: systematic review and meta-analyses based on clinical study reports. BMJ. 2016;352:i65.

42. Chou R, Dana T, Blazina I, et al. Statins for prevention of cardiovascular disease in adults: evidence report and systematic review for the US Preventive Services Task Force. JAMA. 2016;316:2008-24.

43. Franco OH, Chowdhury R, Troup J, et al. Use of plant-based therapies and menopausal symptoms: a systematic review and meta-analysis. JAMA. 2016;315:2554-63.

44. Khera R, Murad MH, Chandar AK, et al. Association of pharmacological treatments for obesity with weight loss and adverse events: a systematic review and meta-analysis. JAMA. 2016;315:2424-34.

45. Lotta LA, Sharp SJ, Burgess S, et al. Association between low-density lipoprotein cholesterol-lowering genetic variants and risk of type 2 diabetes: a meta-analysis. JAMA. 2016;316:1383-91.

46. Palmer SC, Mavridis D, Nicolucci A, et al. Comparison of clinical outcomes and adverse events associated with glucose-lowering drugs in patients with type 2 diabetes: a meta-analysis. JAMA. 2016;316:313-24.

47. Silverman MG, Ference BA, Im K, et al. Association between lowering LDL-C and cardiovascular risk reduction among different therapeutic interventions: a systematic review and meta-analysis. JAMA. 2016;316:1289-97.

48. Abdel Shaheed C, Maher CG, Williams KA, et al. Efficacy, Tolerability, and Dose-Dependent Effects of Opioid Analgesics for Low Back Pain: A Systematic Review and Meta-analysis. JAMA Intern Med. 2016;176:958-68.

49. Jaspers L, Feys F, Bramer WM, et al. Efficacy and Safety of Flibanserin for the Treatment of Hypoactive Sexual Desire Disorder in Women: A Systematic Review and Meta-analysis. JAMA Intern Med. 2016;176:453-62.

50. Thakkar J, Kurup R, Laba TL, et al. Mobile Telephone Text Messaging for Medication Adherence in Chronic Disease: A Meta-analysis. JAMA Intern Med. 2016;176:340-9.

51. Cassese S, Byrne RA, Ndrepepa G, et al. Everolimus-eluting bioresorbable vascular scaffolds versus everolimus-eluting metallic stents: a meta-analysis of randomised controlled trials. Lancet. 2016;387:537-44.

52. Cipriani A, Zhou X, Del Giovane C, et al. Comparative efficacy and tolerability of antidepressants for major depressive disorder in children and adolescents: a network meta-analysis. Lancet. 2016;388:881-90.

53. da Costa BR, Reichenbach S, Keller N, et al. Effectiveness of non-steroidal anti-inflammatory drugs for the treatment of pain in knee and hip osteoarthritis: a network meta-analysis. Lancet. 2016;387:2093-105.

54. Ettehad D, Emdin CA, Kiran A, et al. Blood pressure lowering for prevention of cardiovascular disease and death: a systematic review and meta-analysis. Lancet. 2016;387:957-67.

55. NCD Risk Factor Collaboration (NCD-RisC). Worldwide trends in diabetes since 1980: a pooled analysis of 751 population-based studies with 4.4 million participants. Lancet. 2016;387:1513-30.

56. Rothwell PM, Algra A, Chen Z, et al. Effects of aspirin on risk and severity of early recurrent stroke after transient ischaemic attack and ischaemic stroke: time-course analysis of randomised trials. Lancet. 2016;388:365-75.

57. Stone GW, Gao R, Kimura T, et al. 1-year outcomes with the Absorb bioresorbable scaffold in patients with coronary artery disease: a patient-level, pooled meta-analysis. Lancet. 2016;387:1277-89.

58. Xie X, Atkins E, Lv J, et al. Effects of intensive blood pressure lowering on cardiovascular and renal outcomes: updated systematic review and meta-analysis. Lancet. 2016;387:435-43.

59. Bannuru RR, McAlindon TE, Sullivan MC, et al. Effectiveness and implications of alternative placebo treatments: a systematic review and network meta-analysis of osteoarthritis trials. Ann Intern Med. 2015;163:365-72.

60. Bannuru RR, Schmid CH, Kent DM, et al. Comparative effectiveness of pharmacologic interventions for knee osteoarthritis: a systematic review and network meta-analysis. Ann Intern Med. 2015;162:46-54.

61. Chou R, Hashimoto R, Friedly J, et al. Epidural corticosteroid injections for radiculopathy and spinal stenosis: a systematic review and meta-analysis. Ann Intern Med. 2015;163:373-81.

62. Miligkos M, Bannuru RR, Alkofide H, et al. Leukotriene-receptor antagonists versus placebo in the treatment of asthma in adults and adolescents: a systematic review and meta-analysis. Ann Intern Med. 2015;163:756-67.

63. Navarese EP, Kolodziejczak M, Schulze V, et al. effects of proprotein convertase subtilisin/kexin type 9 antibodies in adults with hypercholesterolemia: a systematic review and meta-analysis. Ann Intern Med. 2015;163:40-51.

64. Siemieniuk RA, Meade MO, Alonso-Coello P, et al. Corticosteroid therapy for patients hospitalized with community-acquired pneumonia: a systematic review and meta-analysis. Ann Intern Med. 2015;163:519-28.

65. Smith ME, Haney E, McDonagh M, et al. Treatment of myalgic encephalomyelitis/chronic fatigue syndrome: a systematic review for a National Institutes of Health Pathways to Prevention Workshop. Ann Intern Med. 2015;162:841-50.

66. Spencer FA, Prasad M, Vandvik PO, et al. Longer- versus shorter-duration dual-antiplatelet therapy after drug-eluting stent placement: a systematic review and meta-analysis. Ann Intern Med. 2015;163:118-26.

67. Sundström J, Arima H, Jackson R, et al. Effects of blood pressure reduction in mild hypertension: a systematic review and meta-analysis. Ann Intern Med. 2015;162:184-91.

68. Uthman OA, Okwundu C, Gbenga K, et al. optimal timing of antiretroviral therapy initiation for HIV-infected adults with newly diagnosed pulmonary tuberculosis: a systematic review and meta-analysis. Ann Intern Med. 2015;163:32-9.

69. Alfirevic Z, Keeney E, Dowswell T, et al. Labour induction with prostaglandins: a systematic review and network meta-analysis. BMJ. 2015;350:h217.

70. Balsells M, García-Patterson A, Solà I, et al. Glibenclamide, metformin, and insulin for the treatment of gestational diabetes: a systematic review and meta-analysis. BMJ. 2015;350:h102.

71. Bolland MJ, Leung W, Tai V, et al. Calcium intake and risk of fracture: systematic review. BMJ. 2015;351:h4580.

72. Conway R, Low C, Coughlan RJ, et al. Methotrexate use and risk of lung disease in psoriasis, psoriatic arthritis, and inflammatory bowel disease: systematic literature review and meta-analysis of randomised controlled trials. BMJ. 2015;350:h1269.

73. Emanuel EJ, Bedarida G, Macci K, et al. Quantifying the risks of non-oncology phase I research in healthy volunteers: meta-analysis of phase I studies. BMJ. 2015;350:h3271.

74. Giacoppo D, Gargiulo G, Aruta P, et al. Treatment strategies for coronary in-stent restenosis: systematic review and hierarchical Bayesian network meta-analysis of 24 randomised trials and 4880 patients. BMJ. 2015;351:h5392.

75. Lenzer J. Why aren't the US Centers for Disease Control and Food and Drug Administration speaking with one voice on flu? BMJ. 2015;350:h658.

76. Li BZ, Threapleton DE, Wang JY, et al. Comparative effectiveness and tolerance of treatments for Helicobacter pylori: systematic review and network meta-analysis. BMJ. 2015;351:h4052.

77. Machado GC, Maher CG, Ferreira PH, et al. Efficacy and safety of paracetamol for spinal pain and osteoarthritis: systematic review and meta-analysis of randomised placebo controlled trials. BMJ. 2015;350:h1225.

78. Navarese EP, Andreotti F, Schulze V, et al. Optimal duration of dual antiplatelet therapy after percutaneous coronary intervention with drug eluting stents: meta-analysis of randomised controlled trials. BMJ. 2015;350:h1618.

79. Thomas KH, Martin RM, Knipe DW, et al. Risk of neuropsychiatric adverse events associated with varenicline: systematic review and meta-analysis. BMJ. 2015;350:h1109.

80. Emdin CA, Rahimi K, Neal B, et al. Blood pressure lowering in type 2 diabetes: a systematic review and meta-analysis. JAMA. 2015;313:603-15.

81. Whiting PF, Wolff RF, Deshpande S, et al. Cannabinoids for medical use: a systematic review and meta-analysis. JAMA. 2015;313:2456-73. Erratum in: JAMA. 2016;315:1522. JAMA. 2015;314:2308. JAMA. 2015;314:520. JAMA. 2015;314:837.

82. Di Bona D, Plaia A, Leto-Barone MS, et al. Efficacy of Grass Pollen Allergen Sublingual Immunotherapy Tablets for Seasonal Allergic Rhinoconjunctivitis: A Systematic Review and Meta-analysis. JAMA Intern Med. 2015;175:1301-9.

83. Prasad V, Kim C, Burotto M, et al. The Strength of Association Between Surrogate End Points and Survival in Oncology: A Systematic Review of Trial-Level Meta-analyses. JAMA Intern Med. 2015;175:1389-98.

84. Viswanathan M, Kahwati LC, Golin CE, et al. Medication therapy management interventions in outpatient settings: a systematic review and meta-analysis. JAMA Intern Med. 2015;175:76-87.

85. Blood Pressure Lowering Treatment Trialists' Collaboration, Ying A, Arima H, et al. Effects of blood pressure lowering on cardiovascular risk according to baseline body-mass index: a meta-analysis of randomised trials. Lancet. 2015;385:867-74.

86. Dobson J, Whitley RJ, Pocock S, et al. Oseltamivir treatment for influenza in adults: a meta-analysis of randomised controlled trials. Lancet. 2015;385:1729-1737. Erratum in: Lancet. 2015;385:1728. Lancet. 2015;385:1728.

87. Early Breast Cancer Trialists' Collaborative Group (EBCTCG). Aromatase inhibitors versus tamoxifen in early breast cancer: patient-level meta-analysis of the randomised trials. Lancet. 2015;386:1341-52.

88. Early Breast Cancer Trialists' Collaborative Group (EBCTCG). Adjuvant bisphosphonate treatment in early breast cancer: meta-analyses of individual patient data from randomised trials. Lancet. 2015;386:1353-61. Erratum in: Lancet. 2016;387:30.

89. Elmariah S, Mauri L, Doros G, et al. Extended duration dual antiplatelet therapy and mortality: a systematic review and meta-analysis. Lancet. 2015;385:792-98. Erratum in: Lancet. 2015;385:1834.

90. Hole J, Hirsch M, Ball E, et al. Music as an aid for postoperative recovery in adults: a systematic review and meta-analysis. Lancet. 2015;386:1659-1671. Erratum in: Lancet. 2015;386:1630.

91. Mega JL, Stitziel NO, Smith JG, et al. Genetic risk, coronary heart disease events, and the clinical benefit of statin therapy: an analysis of primary and secondary prevention trials. Lancet. 2015;385:2264-71.

92. Palmer SC, Mavridis D, Navarese E, et al. Comparative efficacy and safety of blood pressure-lowering agents in adults with diabetes and kidney disease: a network meta-analysis. Lancet. 2015;385:2047-56.

93. Palmerini T, Benedetto U, Bacchi-Reggiani L, et al. Mortality in patients treated with extended duration dual antiplatelet therapy after drug-eluting stent implantation: a pairwise and Bayesian network meta-analysis of randomised trials. Lancet. 2015;385:2371-82.

94. Singh JA, Cameron C, Noorbaloochi S, et al. Risk of serious infection in biological treatment of patients with rheumatoid arthritis: a systematic review and meta-analysis. Lancet. 2015;386:258-65.

95. Siontis GC, Stefanini GG, Mavridis D, et al. Percutaneous coronary interventional strategies for treatment of in-stent restenosis: a network meta-analysis. Lancet. 2015;386:655-64.

96. Swerdlow DI, Preiss D, Kuchenbaecker KB, et al. HMG-coenzyme A reductase inhibition, type 2 diabetes, and bodyweight: evidence from genetic analysis and randomised trials. Lancet. 2015;385:351-61.

97. Viale L, Allotey J, Cheong-See F, et al. Epilepsy in pregnancy and reproductive outcomes: a systematic review and meta-analysis. Lancet. 2015;386:1845-52.

98. Al-Khatib SM, Allen LaPointe NM, et al. Rate- and rhythm-control therapies in patients with atrial fibrillation: a systematic review. Ann Intern Med. 2014;160:760-73.

99. Danese S, Fiorino G, Peyrin-Biroulet L, et al. Biological agents for moderately to severely active ulcerative colitis: a systematic review and network meta-analysis. Ann Intern Med. 2014;160:704-11.

100. Griebeler ML, Morey-Vargas OL, Brito JP, et al. Pharmacologic interventions for painful diabetic neuropathy: An umbrella systematic review and comparative effectiveness network meta-analysis. Ann Intern Med. 2014;161:639-49. Erratum in: Ann Intern Med. 2015;162:600. Ann Intern Med. 2015;162:739.

101. Shaw RJ, McDuffie JR, Hendrix CC, et al. Effects of nurse-managed protocols in the outpatient management of adults with chronic conditions: a systematic review and meta-analysis. Ann Intern Med. 2014;161:113-21.

102. Stagg HR, Zenner D, Harris RJ, et al. Treatment of latent tuberculosis infection: a network meta-analysis. Ann Intern Med. 2014;161:419-28.

103. Tu B, Rich B, Labos C, et al. Coronary revascularization in diabetic patients: a systematic review and Bayesian network meta-analysis. Ann Intern Med. 2014;161:724-32.

104. Bramham K, Parnell B, Nelson-Piercy C, et al. Chronic hypertension and pregnancy outcomes: systematic review and meta-analysis. BMJ. 2014;348:g2301.

105. Ford AC, Forman D, Hunt RH, et al. Helicobacter pylori eradication therapy to prevent gastric cancer in healthy asymptomatic infected individuals: systematic review and meta-analysis of randomised controlled trials. BMJ. 2014;348:g3174.

106. Gough EK, Moodie EE, Prendergast AJ, et al. The impact of antibiotics on growth in children in low and middle income countries: systematic review and meta-analysis of randomised controlled trials. BMJ. 2014;348:g2267.

107. Jefferson T, Jones M, Doshi P, et al. Oseltamivir for influenza in adults and children: systematic review of clinical study reports and summary of regulatory comments. BMJ. 2014;348:g2545.

108. Keene D, Price C, Shun-Shin MJ, et al. Effect on cardiovascular risk of high density lipoprotein targeted drug treatments niacin, fibrates, and CETP inhibitors: meta-analysis of randomised controlled trials including 117,411 patients. BMJ. 2014;349:g4379.

109. Knoll GA, Kokolo MB, Mallick R, et al. Effect of sirolimus on malignancy and survival after kidney transplantation: systematic review and meta-analysis of individual patient data. BMJ. 2014;349:g6679. Erratum in: BMJ. 2014;349:g7543.

110. Loymans RJ, Gemperli A, Cohen J, et al. Comparative effectiveness of long term drug treatment strategies to prevent asthma exacerbations: network meta-analysis. BMJ. 2014;348:g3009.

111. Naci H, Dias S, Ades AE. Industry sponsorship bias in research findings: a network meta-analysis of LDL cholesterol reduction in randomised trials of statins. BMJ. 2014;349:g5741.

112. Taylor D, Sparshatt A, Varma S, et al. Antidepressant efficacy of agomelatine: meta-analysis of published and unpublished studies. BMJ. 2014;348:g1888. Erratum in: BMJ. 2014;348:g2496.

113. Tricco AC, Ashoor HM, Antony J, et al. Safety, effectiveness, and cost effectiveness of long acting versus intermediate acting insulin for patients with type 1 diabetes: systematic review and network meta-analysis. BMJ. 2014;349:g5459.

114. Valgimigli M, Sabaté M, Kaiser C, et al. Effects of cobalt-chromium everolimus eluting stents or bare metal stent on fatal and non-fatal cardiovascular events: patient level meta-analysis. BMJ. 2014;349:g6427.

115. Windecker S, Stortecky S, Stefanini GG, et al. Revascularisation versus medical treatment in patients with stable coronary artery disease: network meta-analysis. BMJ. 2014;348:g3859. Erratum in: BMJ. 349:g4605.

116. Coresh J, Turin TC, Matsushita K, et al. Decline in estimated glomerular filtration rate and subsequent risk of end-stage renal disease and mortality. JAMA. 2014;311:2518-31.

117. Castellucci LA, Cameron C, Le Gal G, et al. Clinical and safety outcomes associated with treatment of acute venous thromboembolism: a systematic review and meta-analysis. JAMA. 2014;312:1122-35.

118. Chatterjee S, Chakraborty A, Weinberg I, et al. Thrombolysis for pulmonary embolism and risk of all-cause mortality, major bleeding, and intracranial hemorrhage: a meta-analysis. JAMA. 2014;311:2414-21.

119. Jonas DE, Amick HR, Feltner C, et al. Pharmacotherapy for adults with alcohol use disorders in outpatient settings: a systematic review and meta-analysis. JAMA. 2014;311:1889-900.

120. Lee JK, Hahn S, Kim DW, et al. Epidermal growth factor receptor tyrosine kinase inhibitors vs conventional chemotherapy in non-small cell lung cancer harboring wild-type epidermal growth factor receptor: a meta-analysis. JAMA. 2014;311:1430-7.

121. Sachar H, Vaidya K, Laine L. Intermittent vs continuous proton pump inhibitor therapy for high-risk bleeding ulcers: a systematic review and meta-analysis. JAMA Intern Med. 2014;174:1755-62.

122. Zusterzeel R, Selzman KA, Sanders WE, et al. Cardiac resynchronization therapy in women: US Food and Drug Administration meta-analysis of patient-level data. JAMA Intern Med. 2014;174:1340-8.

123. Cheng J, Zhang W, Zhang X, et al. Effect of angiotensin-converting enzyme inhibitors and angiotensin II receptor blockers on all-cause mortality, cardiovascular deaths, and cardiovascular events in patients with diabetes mellitus: a meta-analysis. JAMA Intern Med. 2014;174:773-85.

124. Sipahi I, Akay MH, Dagdelen S, et al. Coronary artery bypass grafting vs percutaneous coronary intervention and long-term mortality and morbidity in multivessel disease: meta-analysis of randomized clinical trials of the arterial grafting and stenting era. JAMA Intern Med. 2014;174:223-30.

125. Sipahi I, Celik S, Tozun N. A comparison of results of the US food and drug administration's mini-sentinel program with randomized clinical trials: the case of gastrointestinal tract bleeding with dabigatran. JAMA Intern Med. 2014;174:150-1.

126. Blood Pressure Lowering Treatment Trialists' Collaboration, Sundström J, Arima H, et al. Blood pressure-lowering treatment based on cardiovascular risk: a meta-analysis of individual patient data. Lancet. 2014;384:591-8.

127. Cortazar P, Zhang L, Untch M, et al. Pathological complete response and long-term clinical benefit in breast cancer: the CTNeoBC pooled analysis. Lancet. 2014;384:164-72.

128. Emberson J, Lees KR, Lyden P, et al. Effect of treatment delay, age, and stroke severity on the effects of intravenous thrombolysis with alteplase for acute ischaemic stroke: a meta-analysis of individual patient data from randomised trials. Lancet. 2014;384:1929-35.

129. Eng C, Kramer CK, Zinman B, et al. Glucagon-like peptide-1 receptor agonist and basal insulin combination treatment for the management of type 2 diabetes: a systematic review and meta-analysis. Lancet. 2014;384:2228-34.

130. Kotecha D, Holmes J, Krum H, et al. Efficacy of β blockers in patients with heart failure plus atrial fibrillation: an individual-patient data meta-analysis. Lancet. 2014;384:2235-43.

131. NSCLC Meta-analysis Collaborative Group. Preoperative chemotherapy for non-small-cell lung cancer: a systematic review and meta-analysis of individual participant data. Lancet. 2014;383:1561-71.

132. Reid IR, Bolland MJ, Grey A. Effects of vitamin D supplements on bone mineral density: a systematic review and meta-analysis. Lancet. 2014;383:146-55.

133. Ruff CT, Giugliano RP, Braunwald E, et al. Comparison of the efficacy and safety of new oral anticoagulants with warfarin in patients with atrial fibrillation: a meta-analysis of randomised trials. Lancet. 2014;383:955-62.

134. D'Ambrosio L, Aglietta M, Grignani G. Anticoagulation for central venous catheters in patients with cancer. N Engl J Med. 2014;371:1362-3.

135. Fu R, Selph S, McDonagh M, et al. Effectiveness and harms of recombinant human bone morphogenetic protein-2 in spine fusion: a systematic review and meta-analysis. Ann Intern Med. 2013;158:890-902.

136. Lee M, Saver JL, Hong KS, et al. Risk-benefit profile of long-term dual- versus single-antiplatelet therapy among patients with ischemic stroke: a systematic review and meta-analysis. Ann Intern Med. 2013;159:463-70.

137. Morgan RL, Baack B, Smith BD, et al. Eradication of hepatitis C virus infection and the development of hepatocellular carcinoma: a meta-analysis of observational studies. Ann Intern Med. 2013;158:329-37.

138. Raman G, Moorthy D, Hadar N, et al. Management strategies for asymptomatic carotid stenosis: a systematic review and meta-analysis. Ann Intern Med. 2013;158:676-85.

139. Vasilakou D, Karagiannis T, Athanasiadou E, et al. Sodium-glucose cotransporter 2 inhibitors for type 2 diabetes: a systematic review and meta-analysis. Ann Intern Med. 2013;159:262-74.

140. Azad MB, Coneys JG, Kozyrskyj AL, et al. Probiotic supplementation during pregnancy or infancy for the prevention of asthma and wheeze: systematic review and meta-analysis. BMJ. 2013;347:f6471.

141. Bangalore S, Toklu B, Amoroso N, et al. Bare metal stents, durable polymer drug eluting stents, and biodegradable polymer drug eluting stents for coronary artery disease: mixed treatment comparison meta-analysis. BMJ. 2013;347:f6625.

142. Bermingham SL, Hodgkinson S, Wright S, et al. Intermittent self catheterisation with hydrophilic, gel reservoir, and non-coated catheters: a systematic review and cost effectiveness analysis. BMJ. 2013;346:e8639.

143. Blood Pressure Lowering Treatment Trialists' Collaboration, Ninomiya T, Perkovic V, et al. Blood pressure lowering and major cardiovascular events in people with and without chronic kidney disease: meta-analysis of randomized controlled trials. BMJ. 2013;347:f5680.

144. Chatterjee S, Biondi-Zoccai G, Abbate A, et al. Benefits of β blockers in patients with heart failure and reduced ejection fraction: network meta-analysis. BMJ. 2013;346:f55. Erratum in: BMJ. 2013;346:f596.

145. Cipriani A, Hawton K, Stockton S, et al. Lithium in the prevention of suicide in mood disorders: updated systematic review and meta-analysis. BMJ. 2013;346:f3646.

146. Dormuth CR, Hemmelgarn BR, Paterson JM, et al. Use of high potency statins and rates of admission for acute kidney injury: multicenter, retrospective observational analysis of administrative databases. BMJ. 2013;346:f880.

147. Gagne JJ, Bykov K, Choudhry NK, et al. Effect of smoking on comparative efficacy of antiplatelet agents: systematic review, meta-analysis, and indirect comparison. BMJ. 2013;347:f5307.

148. Gatta L, Vakil N, Vaira D, et al. Global eradication rates for Helicobacter pylori infection: systematic review and meta-analysis of sequential therapy. BMJ. 2013;347:f4587.

149. Makani H, Bangalore S, Desouza KA, et al. Efficacy and safety of dual blockade of the renin-angiotensin system: meta-analysis of randomized trials. BMJ. 2013;346:f360.

150. Marschall J, Carpenter CR, Fowler S, et al. Antibiotic prophylaxis for urinary tract infections after removal of urinary catheter: meta-analysis. BMJ. 2013;346:f3147. Erratum in: BMJ. 2013;347:f5325.

151. Naci H, Ioannidis JP. Comparative effectiveness of exercise and drug interventions on mortality outcomes: metaepidemiological study. BMJ. 2013;347:f5577.

152. Navarese EP, Tandjung K, Claessen B, et al. Safety and efficacy outcomes of first and second generation durable polymer drug eluting stents and biodegradable polymer biolimus eluting stents in clinical practice: comprehensive network meta-analysis. BMJ. 2013;347:f6530.

153. Wu HY, Huang JW, Lin HJ, et al. Comparative effectiveness of renin-angiotensin system blockers and other antihypertensive drugs in patients with diabetes: systematic review and bayesian network meta-analysis. BMJ. 2013;347:f6008.

154. Kayentao K, Garner P, van Eijk AM, et al. Intermittent preventive therapy for malaria during pregnancy using 2 vs 3 or more doses of sulfadoxine-pyrimethamine and risk of low birth weight in Africa: systematic review and meta-analysis. JAMA. 2013;309:594-604.

155. Mead GE, Hsieh CF, Hackett M. Selective serotonin reuptake inhibitors for stroke recovery. JAMA. 2013;310:1066-7.

156. Meissner K, Fässler M, Rücker G, et al. Differential effectiveness of placebo treatments: a systematic review of migraine prophylaxis. JAMA Intern Med. 2013;173:1941-51.

157. Wilt TJ, MacDonald R, Ouellette J, et al. Pharmacologic therapy for primary restless legs syndrome: a systematic review and meta-analysis. JAMA Intern Med. 2013;173:496-505.

158. Coxib and traditional NSAID Trialists' (CNT) Collaboration, Bhala N, Emberson J, et al. Vascular and upper gastrointestinal effects of non-steroidal anti-inflammatory drugs: meta-analyses of individual participant data from randomised trials. Lancet. 2013;382:769-79.

159. Cuzick J, Sestak I, Bonanni B, et al. Selective oestrogen receptor modulators in prevention of breast cancer: an updated meta-analysis of individual participant data. Lancet. 2013;381:1827-34.

160. Leucht S, Cipriani A, Spineli L, et al. Comparative efficacy and tolerability of 15 antipsychotic drugs in schizophrenia: a multiple-treatments meta-analysis. Lancet. 2013;382:951-62.

161. Stefanini GG, Baber U, Windecker S, et al. Safety and efficacy of drug-eluting stents in women: a patient-level pooled analysis of randomised trials. Lancet. 2013;382:1879-88.

162. Tusting LS, Willey B, Lucas H, et al. Socioeconomic development as an intervention against malaria: a systematic review and meta-analysis. Lancet. 2013;382:963-72.

163. Johnston BC, Ma SS, Goldenberg JZ, et al. Probiotics for the prevention of Clostridium difficile-associated diarrhea: a systematic review and meta-analysis. Ann Intern Med. 2012;157:878-88.

164. Pinto RZ, Maher CG, Ferreira ML, et al. Epidural corticosteroid injections in the management of sciatica: a systematic review and meta-analysis. Ann Intern Med. 2012;157:865-77.

165. Rutjes AW, Jüni P, da Costa BR, et al. Viscosupplementation for osteoarthritis of the knee: a systematic review and meta-analysis. Ann Intern Med. 2012;157:180-91.

166. Yeh HC, Brown TT, Maruthur N, et al. Comparative effectiveness and safety of methods of insulin delivery and glucose monitoring for diabetes mellitus: a systematic review and meta-analysis. Ann Intern Med. 2012;157:336-347.

167. Asbridge M, Hayden JA, Cartwright JL. Acute cannabis consumption and motor vehicle collision risk: systematic review of observational studies and meta-analysis. BMJ. 2012;344:e536.

168. Bangalore S, Kumar S, Fusaro M, et al. Outcomes with various drug eluting or bare metal stents in patients with diabetes mellitus: mixed treatment comparison analysis of 22,844 patient years of follow-up from randomised trials. BMJ. 2012;345:e5170.

169. Farmer AJ, Perera R, Ward A, et al. Meta-analysis of individual patient data in randomised trials of self monitoring of blood glucose in people with non-insulin treated type 2 diabetes. BMJ. 2012;344:e486.

170. Fox BD, Kahn SR, Langleben D, et al. Efficacy and safety of novel oral anticoagulants for treatment of acute venous thromboembolism: direct and adjusted indirect meta-analysis of randomised controlled trials. BMJ. 2012;345:e7498.

171. Fürst T, Sayasone S, Odermatt P, et al. Manifestation, diagnosis, and management of foodborne trematodiasis. BMJ. 2012;344:e4093.

172. Haas DM, Caldwell DM, Kirkpatrick P, et al. Tocolytic therapy for preterm delivery: systematic review and network meta-analysis. BMJ. 2012;345:e6226.

173. Harel Z, Gilbert C, Wald R, et al. The effect of combination treatment with aliskiren and blockers of the renin-angiotensin system on hyperkalaemia and acute kidney injury: systematic review and meta-analysis. BMJ. 2012;344:e42.

174. Hemmingsen B, Christensen LL, Wetterslev J, et al. Comparison of metformin and insulin versus insulin alone for type 2 diabetes: systematic review of randomised clinical trials with meta-analyses and trial sequential analyses. BMJ. 2012;344:e1771.

175. Huedo-Medina TB, Kirsch I, Middlemass J, et al. Effectiveness of non-benzodiazepine hypnotics in treatment of adult insomnia: meta-analysis of data submitted to the Food and Drug Administration. BMJ. 2012;345:e8343.

176. Karagiannis T, Paschos P, Paletas K, et al. Dipeptidyl peptidase-4 inhibitors for treatment of type 2 diabetes mellitus in the clinical setting: systematic review and meta-analysis. BMJ. 2012;344:e1369.

177. Low EV, Avery AJ, Gupta V, et al. Identifying the lowest effective dose of acetazolamide for the prophylaxis of acute mountain sickness: systematic review and meta-analysis. BMJ. 2012;345:e6779.

178. Pinto RZ, Maher CG, Ferreira ML, et al. Drugs for relief of pain in patients with sciatica: systematic review and meta-analysis. BMJ. 2012;344:e497.

179. Prochaska JJ, Hilton JF. Risk of cardiovascular serious adverse events associated with varenicline use for tobacco cessation: systematic review and meta-analysis. BMJ. 2012;344:e2856.

180. Vilsbøll T, Christensen M, Junker AE, et al. Effects of glucagon-like peptide-1 receptor agonists on weight loss: systematic review and meta-analyses of randomised controlled trials. BMJ. 2012;344:d7771.

181. Boekholdt SM, Arsenault BJ, Mora S, et al. Association of LDL cholesterol, non-HDL cholesterol, and apolipoprotein B levels with risk of cardiovascular events among patients treated with statins: a meta-analysis. JAMA. 2012;307:1302-9. Erratum in: JAMA. 2012;307:1915. JAMA. 2012;307:1694.

182. Jackson JL, Kuriyama A, Hayashino Y. Botulinum toxin A for prophylactic treatment of migraine and tension headaches in adults: a meta-analysis. JAMA. 2012;307:1736-45.

183. Lopez-Olivo MA, Tayar JH, Martinez-Lopez JA, et al. Risk of malignancies in patients with rheumatoid arthritis treated with biologic therapy: a meta-analysis. JAMA. 2012;308:898-908.

184. Preiss D, Tikkanen MJ, Welsh P, et al. Lipid-modifying therapies and risk of pancreatitis: a meta-analysis. JAMA. 2012;308:804-11.

185. Crawley J, Sismanidis C, Goodman T, et al. Effect of intermittent preventive treatment for malaria during infancy on serological responses to measles and other vaccines used in the Expanded Programme on Immunization: results from five randomised controlled trials. Lancet. 2012;380:1001-10.

186. Early Breast Cancer Trialists' Collaborative Group (EBCTCG), Peto R, Davies C, et al. Comparisons between different polychemotherapy regimens for early breast cancer: meta-analyses of long-term outcome among 100,000 women in 123 randomised trials. Lancet. 2012;379:432-44.

187. Heneghan C, Ward A, Perera R, et al. Self-monitoring of oral anticoagulation: systematic review and meta-analysis of individual patient data. Lancet. 2012;379:322-34.

188. Interleukin-6 Receptor Mendelian Randomisation Analysis (IL6R MR) Consortium, Swerdlow DI, Holmes MV, et al. The interleukin-6 receptor as a target for prevention of coronary heart disease: a mendelian randomisation analysis. Lancet. 2012;379:1214-24.

189. Leucht S, Tardy M, Komossa K, et al. Antipsychotic drugs versus placebo for relapse prevention in schizophrenia: a systematic review and meta-analysis. Lancet. 2012;379:2063-71.

190. Palmerini T, Biondi-Zoccai G, Della Riva D, et al. Stent thrombosis with drug-eluting and bare-metal stents: evidence from a comprehensive network meta-analysis. Lancet. 2012;379:1393-402.

191. Rothwell PM, Wilson M, Price JF, et al. Effect of daily aspirin on risk of cancer metastasis: a study of incident cancers during randomised controlled trials. Lancet. 2012;379:1591-601.

192. Thiele H, Wöhrle J, Hambrecht R, et al. Intracoronary versus intravenous bolus abciximab during primary percutaneous coronary intervention in patients with acute ST-elevation myocardial infarction: a randomised trial. Lancet. 2012;379:923-31.

193. Tricco AC, Ivers NM, Grimshaw JM, et al. Effectiveness of quality improvement strategies on the management of diabetes: a systematic review and meta-analysis. Lancet. 2012;379:2252-61.

194. Bischoff-Ferrari HA, Willett WC, Orav EJ, et al. A pooled analysis of vitamin D dose requirements for fracture prevention. N Engl J Med. 2012;36:40-9. Erratum in: N Engl J Med. 2012;367:481.
